# Synaptic Maturation at Cortical Projections to the Lateral Amygdala in a Mouse Model of Rett Syndrome

**DOI:** 10.1371/journal.pone.0011399

**Published:** 2010-07-02

**Authors:** Frédéric Gambino, Malik Khelfaoui, Bernard Poulain, Thierry Bienvenu, Jamel Chelly, Yann Humeau

**Affiliations:** 1 Institut des Neurosciences Cellulaires et Intégratives, CNRS UPR 3212 et Université de Strasbourg, Strasbourg, France; 2 Institut Cochin, INSERM Unité 567, CNRS UMR 8104, Paris V, CHU Cochin, Paris, France; 3 Physiologie cellulaire de la synapse, CNRS UMR 5091 et Université de Bordeaux2, Bordeaux, France; INSERM U901, France

## Abstract

Rett syndrome (RTT) is a neuro-developmental disorder caused by loss of function of Mecp2 - methyl-CpG-binding protein 2 - an epigenetic factor controlling DNA transcription. In mice, removal of Mecp2 in the forebrain recapitulates most of behavioral deficits found in global Mecp2 deficient mice, including amygdala-related hyper-anxiety and lack of social interaction, pointing a role of Mecp2 in emotional learning. Yet very little is known about the establishment and maintenance of synaptic function in the adult amygdala and the role of Mecp2 in these processes. Here, we performed a longitudinal examination of synaptic properties at excitatory projections to principal cells of the lateral nucleus of the amygdala (LA) in Mecp2 mutant mice and their wild-type littermates. We first show that during animal life, Cortico-LA projections switch from a tonic to a phasic mode, whereas Thalamo-LA synapses are phasic at all ages. In parallel, we observed a specific elimination of Cortico-LA synapses and a decrease in their ability of generating presynaptic long term potentiation. In absence of Mecp2, both synaptic maturation and synaptic elimination were exaggerated albeit still specific to cortical projections. Surprisingly, associative LTP was unaffected at Mecp2 deficient synapses suggesting that synaptic maintenance rather than activity-dependent synaptic learning may be causal in RTT physiopathology. Finally, because the timing of synaptic evolution was preserved, we propose that some of the developmental effects of Mecp2 may be exerted within an endogenous program and restricted to synapses which maturate during animal life.

## Introduction

RTT, caused by null mutations of Mecp2 gene [1] is characterized by a period of normal postnatal development followed by a regression of motor language and social skills. This delay in symptoms onset possibly relies to the expression of Mecp2 in mature but not in immature neurons [2], but alternatively, may result from a role of a Mecp2-dependent factor, possibly neurotrophins, in experience-driven synaptic maturation, maintenance and plasticity [3]–[5]. Using α-CamKII promoter-driven recombination, forebrain-specific conditional Mecp2 mutant mice were generated [6] and show that a delayed deletion of Mecp2 in the cortex, the amygdala, the hippocampus and the striatum was sufficient to recapitulate most of behavioral deficits found in constitutive knock-outs (KOs) [6]. Recently, the same group reported that the control of DNA transcription mediated by Mecp2 in the basolateral nucleus of the amygdala (BLA) was responsible for anxiety and fear learning deficits observed in RTT mouse models [7]. A particular role of Mecp2 in amygdala structure is further suggested by its severe volume decrease observed in Mecp2 mutant mice (∼40% to compare with ∼25% in total brain) [8], a structure which size normally increase during childhood in humans, with the remarkable exception of autistic patients [9].

To date the role of Mecp2 in the key phases of synaptic life – synaptogenesis, synaptic maintenance and synaptic plasticity - is still conflicting: A dramatic loss of excitatory synapses is observed in Mecp2-deficient primary neuronal cultures, but synaptic marker density appears to be normal in hippocampal slices of symptomatic RTT mice [3]. However, recently, a decrease in the connectivity between layer V cortical neurons was reported in Mecp2 mutant mice [10], but only after symptom occurrence, questioning the role of Mecp2 in the maintenance of synaptic contacts in adult neuronal networks. Also, associative LTP is not systematically affected in Mecp2 deficient preparations [10]–[12], but when defective, is rescued by reintroduction of Mecp2 [13] suggesting that the presence of Mecp2 in the adult is important to maintain synaptic function, possibly due to the activity-dependent control of BDNF production by Mecp2 [14], [15].

Here we examined synaptogenesis, synaptic maintenance and synaptic plasticity at cortical and sub-cortical excitatory projections to the LA in a model of RTT, Mecp2^308/Y^ (KO) mice [16] and their Mecp2^X/Y^ (WT) littermates. Our data shows that after a period of normal development, synaptic elimination and maturation at cortical-LA synapses were exaggerated in absence of Mecp2. Importantly, the other major excitatory projections to the LA, the so-called Thalamo-LA synapses, were unaffected by the absence of Mecp2. Thus, we propose that some of the developmental effects of Mecp2 are exerted within an endogenous program and restricted to synapses which maturate during animal life.

## Results

### Synaptic maturation and elimination at excitatory projections to the LA in WT mice

**Figure 1:**
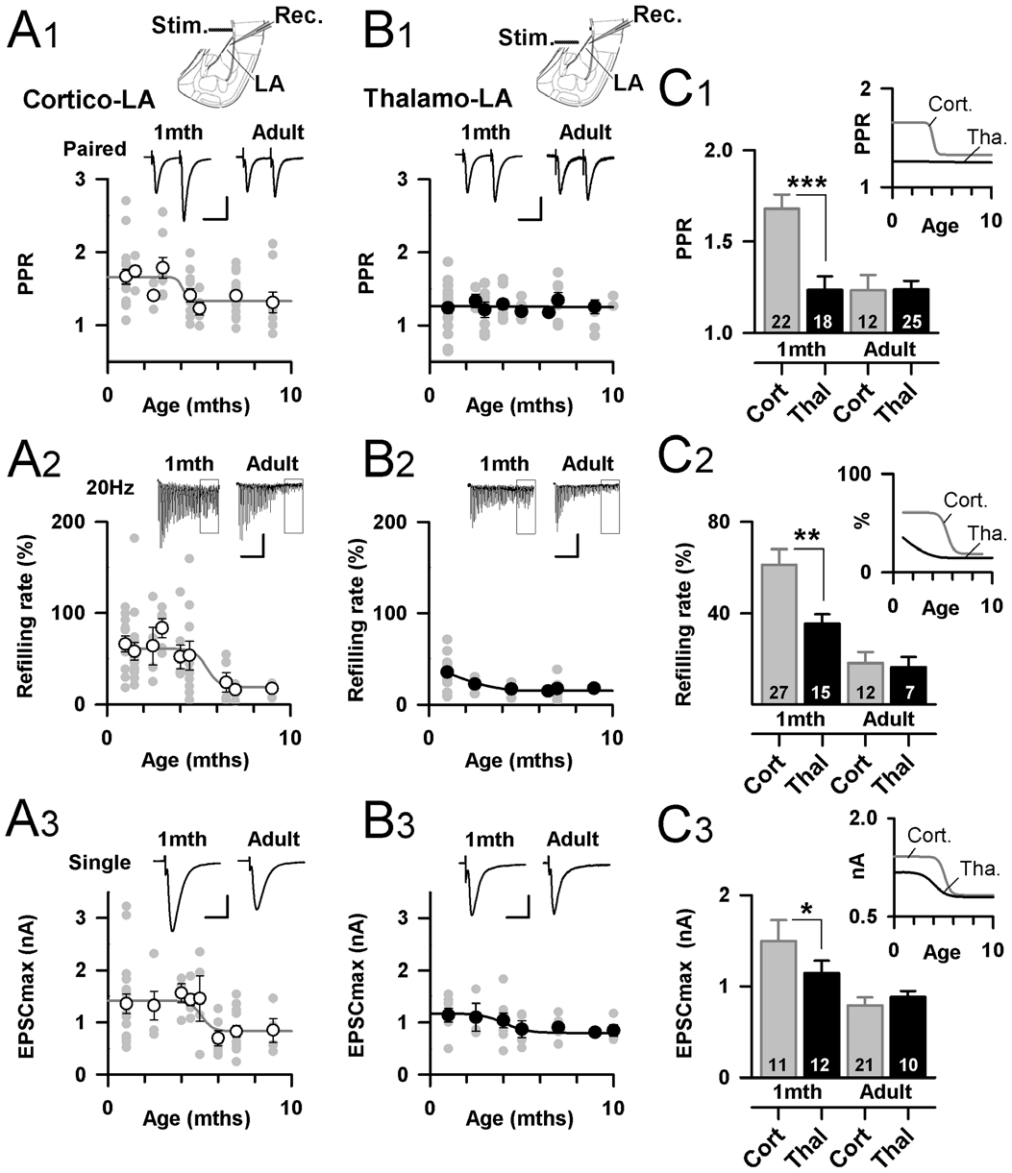
Synaptic maturation and elimination at excitatory projections to the LA in WT mice. A1–3: Developmental time course of paired pulse ratio (PPR), refilling rate and maximal EPSC (EPSCmax) at Cortico-LA synapses. Grey circles represent single experiments and white dots are mean (± SEM) values at a given post-natal age. Top: typical traces. Scale bars: A1: 50 pA and 50 msec, A2: 50 pA and 0.75 sec, A3: 1 nA and 10 msec. A scheme of the experimental preparation is presented on top. B1–3: Same presentation as in A1–3, but for Thalamo-LA synapses. C1–3: Bar graphs summarizing the PPR (C1), refilling rate (C2) and EPSCmax (C3) in juvenile (1 month) and adult (>6 months) WT mice. The number of recorded cells is indicated. *: p<0.05, **: p<0.01, ***: p<0.001. Right: Evolution of synaptic parameters during development.

The amygdala is one of the key brain structures for emotional memory acquisition and storage [17], [18], and substantial evidence supports the notion that Thalamo-LA and Cortico-LA synapses change during fear acquisition [19], [20]. Surprisingly, very few is known about the evolution of the synaptic properties at Cortico-LA and Thalamo-LA synapses during animal life, yet studied in juvenile [21]–[23] or young adult mice [20], [24], [25]. Thus, because in Mecp2^308/Y^ animals symptom onset occurs only after 6–12 weeks of age [16], we first characterized synaptic properties at excitatory projections to the LA in pre-symptomatic (1month old) and post-symptomatic (until 12 months) Mecp2^308/Y^ and their Mecp2^X/Y^ littermates (Fig. 1).

Synaptic physiology at Cortico-LA and Thalamo-LA synapses was examined in acute coronal brain slices using whole-cell patch-clamp recordings from LA principal neurons [21], [26] (Fig. 1). Monosynaptic excitatory postsynaptic currents [EPSCs] were elicited by stimulating cortical axons in the external capsule [20]–[22] (Fig. 1A_1_), and thalamic axons within the internal capsule [26] (Fig. 1B_1_).

We systematically evaluated the global synaptic strength (Input/Output curves, Fig. S1), the paired pulse ratio (PPR) at 50 milliseconds inter-spike intervals [Fig. 1], and the postsynaptic responses to 2 second, 20 Hz stimulations trains, allowing the evaluation of the size of the ready releasable pool [RRP] and the refilling rate [27] (Fig. S2) (Fig. 1). Juvenile Cortico-LA and Thalamo-LA synapses differ in their AMPAR/NMDAR ratio (28, but see 22, 29), the size and molecular equipment of their dendritic spine [22] and the locus of associative plasticity [21], [22], [30]. Here we added to this list a different mode - phasic versus tonic - of neurotransmitter release. Indeed, as shown in Fig. 1B, Juvenile Thalamo-LA synapses typically behave as phasic synapses [31] with a small amount of PPR [1.23±0.07, n = 18 cells] and a low resistance of neurotransmission to high frequency stimulations due to a low refilling rate [35±4%, n = 15, Fig. 1B]. Interestingly, these and other synaptic parameters at these synapses are rather constant during animal life (Fig. 1, Table S1), albeit not excluding some subtle changes such as increased quantal content per axon or decrease in the number of silent synapses which have been reported in juvenile and adult Thalamo-LA synapses in another mouse strain [25].

In contrast, to Thalamo-LA synapses, juvenile Cortico-LA synapses fulfill typical criteria of “Tonic” synapses [31], with high PPR (1.68±0.08, n = 22 cells, Fig. 1C) and high refilling rate (61±7%, n = 27, Fig. 1C). Surprisingly, when assessed in adult animals (>6 months of age), these two parameters evolved in such a way that adult Cortico-LA synapses behave as phasic synapses, similar to their neighboring Thalamo-LA synapses (Fig. 1D). This specific developmental switch in the synaptic modality support an experience-driven maturation of Cortico-LA afferents but not Thalamo-LA synapses during mouse adult life.

In addition to synaptic maturation, we observed a significant synaptic elimination in adult WT animals illustrated by the lower frequency of miniature EPSCs recorded in LA principal cells in presence of the voltage-sensitive sodium channel blocker Tetrodotoxin (TTX, 10 µM) in adult animals (Table S1). As shown in Fig. 1, this may be partially contributed by a loss of functional Cortico-LA synapses as the maximal EPSC in adult is lower than in juvenile animals (Cortico-LA EPSC_max_: 1 month: 1497±225 pA, Adult: 794±82 pA, p<0.001, Fig. 1C). Less synaptic elimination is observed at Thalamo-LA afferents (1 month: 1145±132 pA, Adult: 882±65 pA, p<0.05, Fig. 1C) reinforcing the idea that synaptic maturation at these synapses is almost completed after 1 month of age in mice. All together our findings suggest a strong maturation of Cortico-LA synapses during the mouse adulthood, whereas Thalamo-LA synapses appear globally fixed even in juvenile animals.

### Synaptic maturation and elimination at excitatory projections to the LA in Mecp2 deficient mice

**Figure 2:**
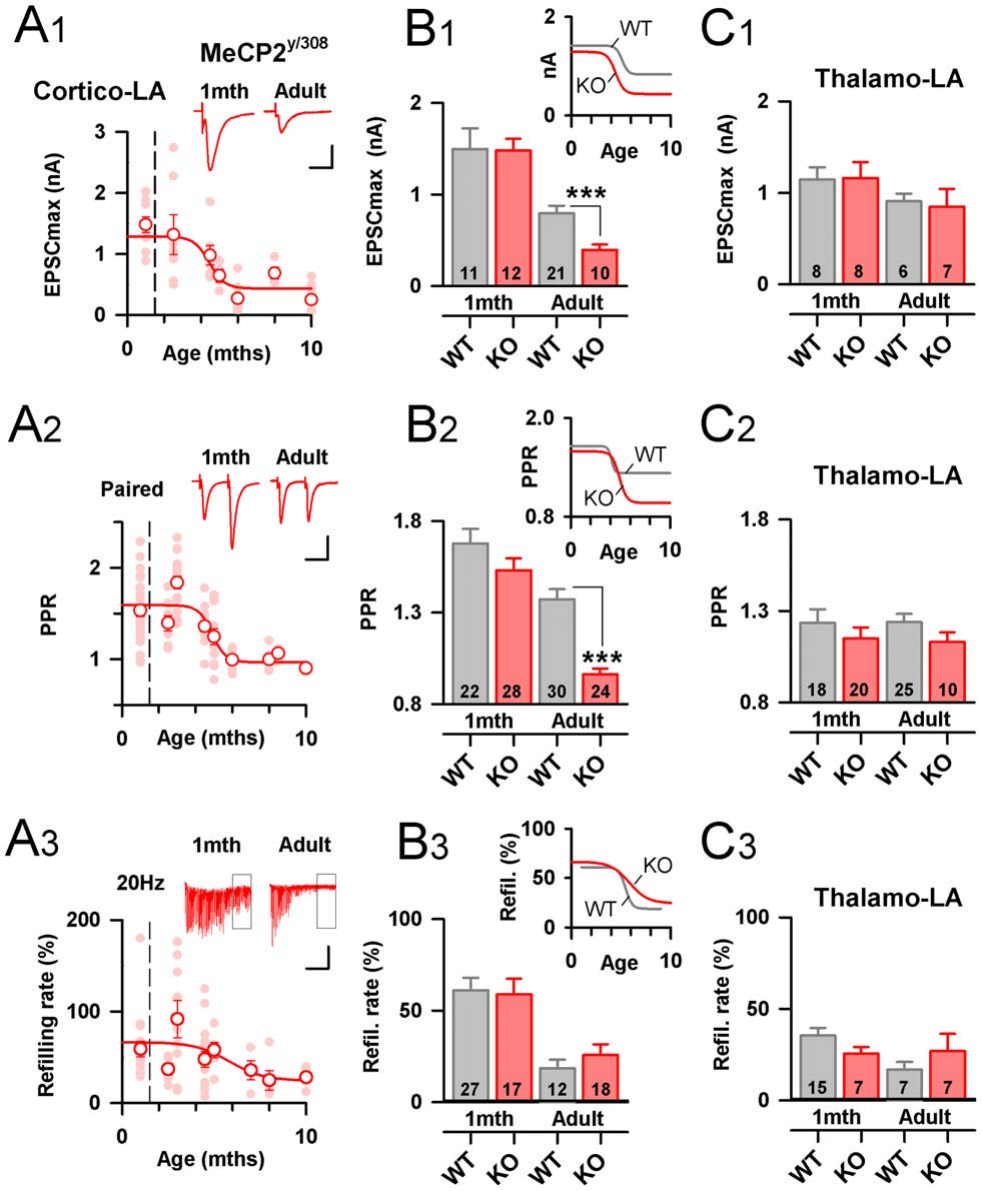
Synaptic maturation and elimination at excitatory projections to the LA in Mecp2 deficient mice. A1–3: Developmental time course of EPSCmax, PPR and refilling rateat Cortico-LA synapses of Mecp2^308/Y^ KO mice. Light red circles represent single experiments and white dots are mean (± SEM) values at a given post-natal age. Top: typical traces. Scale bars: A1: 1 nA and 10 msec. A2: 50 pA and 0.5 sec, A3: 100 pA and 1 sec. B1–3: Bar graphs summarizing the EPSCmax (B1), PPR (B2) and refilling rate (C3) at Cortico-LA synapses in juvenile (1 month) and adult (>6 months) KO mice. The number of recorded cells is indicated. *: p<0.05, ***:p<0.001. Right: Evolution of synaptic parameters during development. C1–3: Same presentation as in B1–3, but for Thalamo-LA synapses.

We then performed the same analysis of synaptic function at Cortico-LA and Thalamo-LA synapses from Mecp2^308/Y^ animals (Fig. 2), and compared the results to the synaptic framework built from the Mecp2^X/Y^ WT littermates (Fig. 1).

First, despite the fact that, in extent, some synaptic parameters change exaggeratedly (see below), the global evolution of Cortico-LA and Thalamo-LA synapses is preserved in absence of Mecp2. In Mecp2^308/Y^ animals, we still observed a switch in the modality of Cortico-LA synapses (Fig. 2). In addition, as in WT mice, Thalamo-LA synapses showed very little maturation during adulthood (Fig. 2C). However, we gain key information about the role of Mecp2 in amygdala development from this comparative longitudinal study: First, all synaptic parameters collected in juvenile animals were independent to the presence or absence of Mecp2. Indeed, in addition to the synapse specific parameters presented in Fig. 2B and 2C, no changes in the frequency and amplitude of miniature EPSCs, in the AMPA/NMDA ratio and RRP size at both excitatory synapses could be detected (Table S1). Further, associative Long term potentiation (LTP) was similar at juvenile Cortico-LA and Thalamo-LA synapses in Mecp2^308/Y^ and Mecp2^X/Y^ animals (Table S1). Thus, our data strongly support the notion that Mecp2 is dispensable for synaptogenesis and synaptic function in the young mouse amygdala. Second, we observed that two of the synaptic parameters developmentally regulated in WT mice were more dramatically affected in adult Mecp2^308/Y^ mice, albeit evolving with a similar timing (EPSC_max_ Adult: WT: 794±82 pA, KO: 392±59 pA, p<0.001; PPR: WT: 1.37±0.05, KO: 0.96±0.03, p<0.001, Fig. 2B). This suggests that both synaptic elimination and maturation at Cortico-LA projections are amplified in absence of Mecp2. Importantly, these findings were confirmed by other sets of data: We first tested minimal stimulations of cortical inputs in old Mecp2^308/Y^ and Mecp2^X/Y^ animals (Fig. S3). In absence of Mecp2, the minimal Cortico-LA response was indeed significantly lower than in WT mice (Fig. S3). In adult Mecp2^308/Y^ mice, the miniature EPSC frequency was also decreased (Table S1) as well as the coefficient of variation at Cortico-LA but not Thalamo-LA synapses (Table S1). Together with the strong decrease in Cortico-LA PPR in adult Mecp2^308/Y^ mice (Fig. 2), we conclude that the prolonged absence of Mecp2 strongly increased release probability at Cortico-LA synapses, possibly compensating for the progressive synaptic elimination.

### Intact associative LTP at Cortico-LA synapses in early and late symptomatic Mecp2^308/Y^ mutant mice

**Figure 3:**
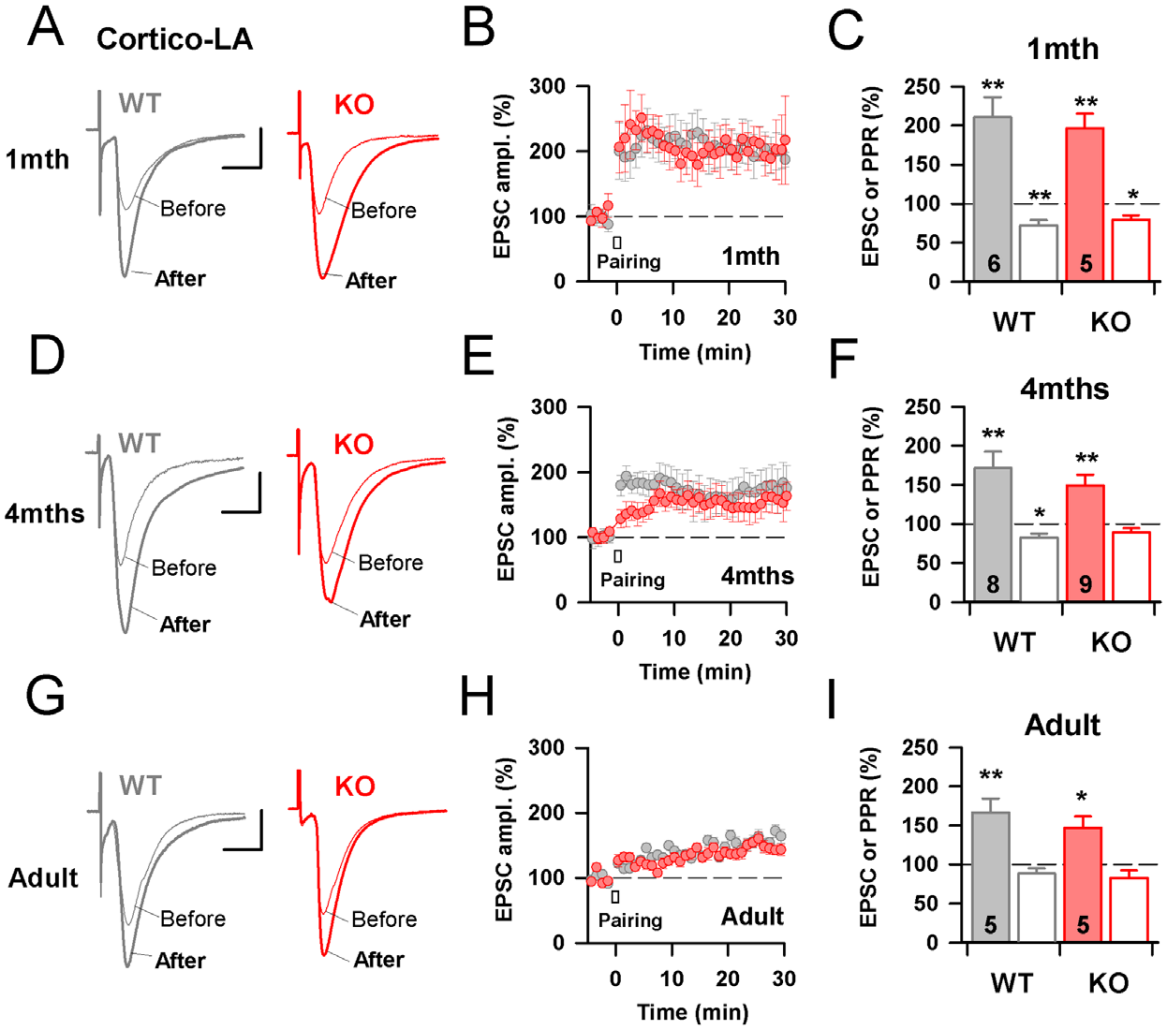
Intact associative LTP at Cortico-LA synapses in early and late symptomatic Mecp2308/Y mutant mice. A: Typical EPSCs obtained in juvenile WT (grey) and KO (red) mice before and after pairing (see material and methods section). Scale bars: 50 pA and 10 msec. B: Mecp2 is not necessary for presynaptic LTP in juvenile mice. C: Bar graphs summarizing the evolution of the EPSC (filled bars) and PPR (empty bars) in WT (grey) and KO (red) mice. The number of recorded cells is indicated. *: p<0.05, **: p<0.01. D–F: Same presentation as in A–C, but for 4 months old WT and KO animals. G–I: Same presentation as in A–C, but for adult (>6 months) WT and KO animals.

Next, we examined associative LTP at Cortico-LA synapses of adult Mecp2 KO mice. Indeed, it is now well established that like its counterparts in other brain areas [32]–[36], LTP at Cortico-LA synapses is predominantly expressed by a presynaptic, cAMP/PKA-dependent increase in the probability of release [20], [21], [23], [37]–[38]. Thus, if the absence of Mecp2 modified release probability by the mean of a PKA-dependent mechanism, a saturation of presynaptic LTP expression may be expected in adult Mecp2^308/Y^ animals. Accordingly to earlier studies reporting that changes in release probability at Cortico-LA synapses were consecutive to fear learning [20], the amount of presynaptic associative LTP was lower in adult WT animals as compared to juveniles (WT 1month: 210±26% of baseline, WT Adult: 167±18% of baseline, p<0.05, Fig. 3). Importantly, at each tested post natal age the absence of Mecp2 never impacts associative presynaptic LTP (KO Adult: 147±15% of baseline, p>0.05 as compared to WT adult, and p<0.05 as compared to baseline, Fig. 3) suggesting that the increase in release probability at Mecp2 deficient adult Cortico-LA synapses may not be linked to an over-activation of the cAMP/PKA/Rim pathway [38]. This hypothesis was confirmed by pharmacological activations of adenylate cyclase (AC) by Forskolin (50 µM), a condition which we used previously in juvenile animals to demonstrate the role of the cAMP/PKA/Rim in the control of release probability at Cortico-LA synapses [38]. Indeed, when applied on 10–12 months old adult mice preparations, Forskolin failed in exerting its effect on evoked synaptic transmission in both WT and KO preparations (Fig.S4), providing a plausible explanation to the progressive decrease of presynaptic LTP levels with age in both genotypes. It also indicates that some qualitative aspects of fear learning, such as generalization [39], [40], may be preserved in late symptomatic RTT mouse models.

### Synaptic elimination is restricted to the AMPA component at Cortico-LA synapses

Next, we examined if the specific Cortico-LA synapse elimination changed the feed-forward recruitment of local GABAergic interneurons, a key physiological parameter for LA function [41]. First, we tested the possibility of a specific loss of AMPAR at Cortico-LA synapses. Indeed, in WT mice we observed a developmental regulation in the NMDA/AMPA ratio at these synapses (Table S1). In Mecp2 mutant mice however, this modulation does not seem to occur, the NMDA/AMPA ratio remaining constant during the animal life (Table S1). Together with the strong decrease of the EPSC_max_ in Mecp2^308/Y^ animals (Fig. 2), these data support a role for Mecp2 in stabilizing AMPAR within Cortico-LA post-synapses.

**Figure 4:**
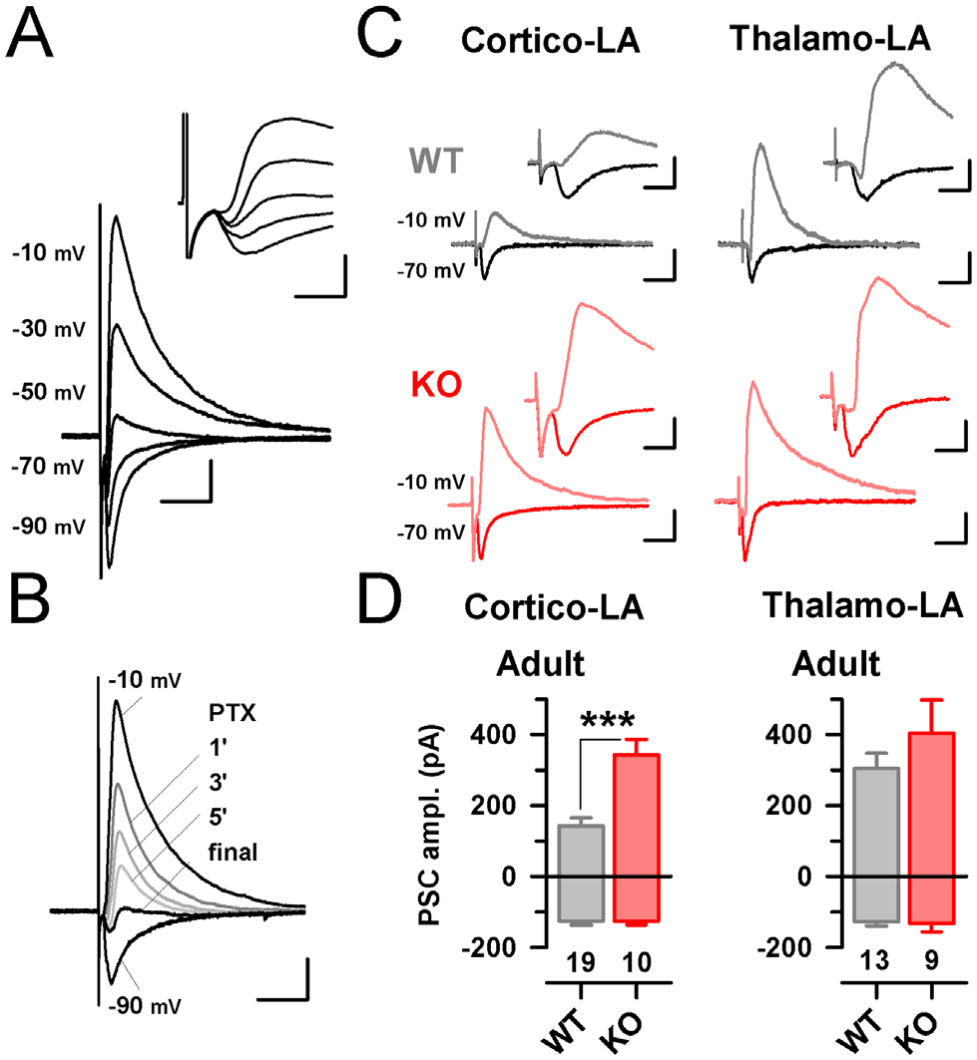
Synaptic elimination is restricted to the AMPA component at Cortico-LA synapses. A: Typical traces obtained at Cortico-LA synapses at various membrane potentials (indicated on the left). Scale bars: 100 pA and 20 msec. Top inset: enlargement of the recording. Note that the outward current is delayed of few milliseconds. Scale bars: 200 pA and 7 msec. B: Typical synaptic responses obtained in absence (black line) and presence of 100 µM Picrotoxin (grey lines). Note the complete blockade of the outward current at −10mV membrane potential. Scale bars: 150 pA and 40 msec. C: Left: typical synaptic currents recorded at −70 and −10 mV at Cortico-LA synapses in adult WT (grey) and KO (red). Right: typical synaptic currents recorded at −70 and −10 mV at Thalamo-LA synapses in adult WT (grey) and KO (red). Scale bars: full traces: 100 pA and 50 msec. Insets: 100 pA and 6 msec. D: Bar graphs summarizing the evolution of the EPSC (bottom bars) and corresponding FF-IPSC (top bars) in WT (grey) and KO (red) mice. The number of recorded cells is indicated. ***: p<0.001.

Next, we wanted to test if the removal of AMPAR could be generalized to other cortical synapses. We previously reported that the feed-forward recruitment of local interneuron was highly depending on AMPAR function at Thalamo-interneuron synapses [26]. Thus we thought to test for the efficacy of incoming glutamatergic inputs to generate GABA_A_-mediated currents in LA principal cells (Fig. 4B). To this aim, excitatory and inhibitory current in principal cells were recorded at −70 mV and −10 mV respectively (Fig. 4B). Interestingly, in young animals both thalamic and cortical afferent we found to be efficient in activating local interneurons [42], [43]. In adult mice however, both excitatory inputs were not equal in recruiting local interneurons: when the stimulation intensity was set to elicit an EPSC of ∼100pA amplitude, a higher IPSC was observed when stimulating within the internal (Thalamo-LA: 304±44 pA, Fig. 4C and 4D) as compared to external capsule [139±29 pA, p<0.001, Fig. 4C and 4D). In absence of Mecp2, a specific increase in feed-forward efficacy was observed at cortical afferents in the adult (259±53 pA, p<0.05, Fig. 4D). These results suggest a specific loss of AMPAR-mediated currents at contacts between cortical afferents and LA principal cells.

## Discussion

Emotional deficits are present in Rett syndrome patients and Mecp2 mutant mice [44], that can be mimicked by specific deletion of Mecp2 within the forebrain [45], [46], and, although only partially, by inactivating Mecp2 in the basolateral amygdala [7]. Thus, because Mecp2 appears to affect synaptic function in many different brain areas [4], we thought to characterize synaptic function at excitatory projections to the LA, required for associative fear learning, perturbed in some Mecp2 mutant mice [46]. However, as compared to other monogenetic causes of mental retardation, behavioral and cognitive symptoms in RTT patients and RTT mouse models are significantly delayed [8]. This introduces the notion of one or more critical windows in the physiopathology of the disease, and therefore forces the establishment of longitudinal frameworks to study synaptic and/or neuronal function in absence of Mecp2. It is noteworthy that most of fear learning studies are performed on adult (3–4 months old animals) whereas most of *in vitro* data are obtained in juvenile animals (<1 month). Thus, in order to reconcile cognitive and physiological data, we examined a broad repertoire of synaptic properties at Cortico-LA and Thalamo-LA synapses in pre-symptomatic (1month old) and post-symptomatic (until 12 months) Mecp2^308/Y^ and their Mecp2^X/Y^ littermates.

Here, we describe a progressive elimination of Cortico-LA starting after 3 months of age and achieved after 6 months (Fig. 1). This process is amplified by the absence of Mecp2, but with a preserved timing (Fig. 2). Other synaptic parameters, such as PPR and refilling rate, evolve similarly, suggesting that this lack of synaptic maintenance may be the end result of synaptic maturation at this excitatory projection. Interestingly, the NMDA/AMPA ratio at these synapses, which progressively decline with age, follows a similar time course (Fig. S5), suggesting that in parallel to synaptic elimination, a reinforcement of AMPAR at subsisting contacts occur. With respect to this, the absence of Mecp2 appears to amplify synaptic elimination without allowing AMPAR redistribution, suggesting a specific role of Mecp2-dependent factors in the maturation of synaptic contacts within the LA.

One intriguing question is the effect of age and of Mecp2 deletion onto Cortico-LA but not Thalamo-LA synapses. Indeed, given the role of Mecp2 in maintaining the integrity of neuronal dendritic arbor [47] one may have expected strong and unspecific rearrangements of synaptic contacts in symptomatic MeCP^308/Y^ mice. However, as reported by Moretti and colleagues, cortical neurons of 12–15 months old Mecp2^308/Y^ animals did not exhibit strong modifications of dendritic arbor or distribution of synaptic markers [12]. Several of our results support this preserved architecture: in late symptomatic - over 6 months old - Mecp2^308/Y^ animals, the EPSC_max_ at Thalamo-LA synapses (Fig. 2), the frequency of spontaneous and amplitude of Thalamo-LA evoked IPSCs were preserved (Fig. S6 and Fig. 4). We also did not detect any difference in the cell capacitance or membrane resistance of LA principal cells according to age or genotype (Table S1). Thus even if some of these arguments can be discussed [25], we think that no global posts-synaptic defect is present in LA principal cells of Mecp2 mutant mice.

Remains the specific effect observed at cortico-LA synapses. One possible explanation is a presynaptic effect of Mecp2 thus pushing towards a role of Mecp2 in the cortex. Indeed, last years, a crucial role of Mecp2 in supporting the BDNF/TRK pathway has been proposed [14], [48]. This hypothesis emerges from several observations linking Mecp2 to BDNF production [14], [15], [48]. In this discussion frame, we can note that BDNF levels are lowered in the cortex of symptomatic animals [14], [48], and that BDNF over-expression can correct for the lack of cortical neuron discharge observed in Mecp2 mutant mice [14]. This is further supported by the fact that changes in PPR and refilling rates are considered to be presynaptic parameters [but see 31, 49]. Thus, even in the absence of any study examining the role of Mecp2 within the thalamus, a specific role of Mecp2 at cortical pre-synapses is plausible. Then another important question comes: Is the amygdala defect presented reflects an essential role of Mecp2 in the LA or a secondary consequence of a dysfunction of neurons projecting to the LA? Indeed, when compared to the pre-symptomatic effects of Mecp2 deletion in the cortex [10], [12], the physiological defects observed in the amygdala are delayed for several months. It is however possible that this difference in timing reside in the expression of delayed endogenous programs, in other words, that the cortex and the amygdala have different critical windows for synaptic maturation. Towards this hypothesis, several longitudinal studies showed that in the mouse, cortical synaptic maturation is achieved after 4–5 weeks of life [50].

There is an alternative to a pure presynaptic – cortical - action of both Mecp2 and age. Indeed, some heterogeneity in the morphology and molecular equipment of post-synaptic compartments at Cortico-LA and Thalamo-LA synapses has been reported in juvenile mice [22], [28], [30]. In a sense, Cortico-LA post-synapses appear rather immature as compared to thalamic ones. Thus one likely possibility is that during animal life cortical dendritic spines maturates, a process which would then depend on the presence of Mecp2.

To conclude, we here show that at excitatory projections to the LA all synaptic effects associated with the absence of Mecp2 were following a developmental time course similar as in WT mice. Thus we propose that some of the developmental effects of Mecp2 are exerted within an endogenous program and restricted to synapses which maturate during animal life.

## Materials and Methods

### Ethic statement

All experiments have been conducted using a protocol approved by the European and French guidelines on animal experimentation. All animal experiments were approved by the direction des services veterinaries du Bas-Rhin, Alsace, France (authorization number 67–313 to YH).

### Animals

Mecp2^308/Y^ and Mecp2^X/Y^ males are from breeding pairs of Mecp2–308 mice backcrossed to C57BL/6J mice. Original mice were from Jackson Laboratories (B6-129S-Mecp2^tm1Hzo/J^, stock number: 005439, USA).

### Slice preparation

Standard procedures were used to prepare 300 µm thick coronal slices from Mecp2^308/Y^ and Mecp2^X/Y^ males on a C57BL/6J background. Briefly, the brain was dissected in ice-cold artificial cerebrospinal fluid (ACSF), mounted on an agar block and sliced with a vibratome (Leica VT1200s; Germany) at 4°C. Slices were maintained for 45 min. at 35°C in an interface chamber containing ACSF equilibrated with 95% O_2_/5% CO_2_ and containing (in mM): 124 NaCl, 2.7 KCl, 2 CaCl_2_, 1.3 MgCl_2_, 26 NaHCO_3_, 0.4 NaH_2_PO_4_, 18 glucose, 4 ascorbate, and then for at least 45 min. at room temperature before being transferred to a superfusing recording chamber.

### Recordings

Whole-cell recordings from LA principal neurons were performed at 30–32°C in a superfusing chamber as previously described [30]. Neurons were visually identified with infrared videomicroscopy using an upright microscope equipped with a 60x objective. Patch electrodes (3–5 MΩ) were pulled from borosilicate glass tubing and filled with a low-chloride solution containing (in mM]: 140 Cs-methylsulfonate, 5 QX314-Cl, 10 HEPES, 10 phosphocreatine, 4 Mg-ATP, and 0.3 Na-GTP [pH adjusted to 7.25 with CsOH, 295 mOsm). All experiments were performed in the presence of picrotoxin [100 µM]. Monosynaptic EPSCs exhibiting constant 10–90% rise times and latencies were elicited by stimulation of afferent fibers with a bipolar twisted platinum/10% iridium wire (25 µm diameter).

### Data acquisition and analysis

Data were recorded with a Multiclamp700B (Molecular Devices, USA), filtered at 2 kHz and digitized at 10 kHz. In all experiments, series resistance was monitored throughout the experiment, and if it changed by more than 15%, the data were not included in the analysis. Data were acquired and analyzed with pClamp10.2 (Molecular Devices). LTP was induced using a pairing protocol consisting of a single burst of presynaptic activity (80 stim at 10 Hz) applied together with a postsynaptic depolarization (+30 mV) during the same 8 sec period. Changes were quantified by normalizing and averaging EPSC amplitudes during the last 5 min. of the experiments relative to the 5 min. of baseline prior to LTP induction or drug application. All values are given as means ± standard error of the mean (SEM). Mean values were compared between genotypes using either unpaired Student's *t*-test or Mann-Whitney (MW) test as appropriate.

### Reagents

Picrotoxin was from Sigma-Aldrich (Saint Quentin Fallavier, France), QX-314 was from Alomone Labs Ltd. (Jerusalem, Israel), and TTX was from Latoxan (Valence, France). Forskolin was from Ascent scientific (Bristol, UK).

## Supporting Information

Table S1 Top: Basic cellular and recording parameters were obtained in juvenile and adult WT and KO mice. The unit of a particular measurement is indicated on the left, and the number of recorded cells in brackets. A blue text means a difference between juveniles and adult WT, and red text between WT and KO at a given postnatal age. Methods table S1 The input resistance (Rinput) and cell capacitance (tau/Rinput) were extracted from 10 mV hyper-polarizing steps in voltage clamp mode. Miniature EPSCs (mEPSCs) were automatically detected by a template-based routine in the ClampFit 10.0 software. Events were then fitted by a standard bi-exponential equation to extract un-noisy amplitude. In general, mEPSC frequency was determined by visual detection (number of peaks) during a 0,5 to 10 minute period depending on the event frequency (a minimum of 200 events was analyzed). The coefficient of variation is determined using at least 30 consecutive EPSCs. No difference between mean EPSC amplitude was detected between considered groups.
(2.92 MB TIF)

Figure S1 Input/Output curves at Thalamo-LA and Cortico-LA synapses. A: Scheme of the experimental preparation. B: Typical EPSCs recorded in LA principal cells following stimulation in the internal [Thalamo-LA] and external [Cortico-LA] capsules in juvenile animals. Stimulation intensities are indicated. Scale bars: 400 pA and 20 msec. C and D: Top: EPSC amplitude obtained at a given stimulation intensity were average and scaled to the maximal EPSC [at 50mA/msec stimulation]. Grey dots: Cortico-LA EPSCs, Black dots: Thalamo-LA EPSCs. In D, stimulation intensities were presented as a logarithmic function to better visualize the difference in efficacy at low stimulations. Bottom: The ratio between normalized EPSCs is presented, showing that Thalamo-LA synapses are more easily activated than Cortico-LA synapses [ratio <1].
(1.10 MB TIF)

Figure S2 Extraction of RRP size and Refilling rate at excitatory projections to the LA. A-C: Response to 20 Hz stimulations at juvenile [white dots] and adult [grey dots] cortico-LA synapses. A: EPSC amplitude at a given position [#1–40] during 20 Hz trains were averaged [n = 27 and 12 cells respectively]. B: Cumulative EPSC amplitude during 20 Hz trains in juvenile [white dots] and adult [grey dots] cortico-LA synapses. Same data as in A. Linear fit were obtained from #30–40. Its slope is an index of the refilling rate, whereas its extension at y = 0 give the size of the readily releasable pool [RRP]. For further details, see [27]. C: Developmental time course of RRP size at Cortico-LA synapses. Grey circles represent single experiments and white dots are mean [± SEM] values at a given post-natal age. Grey line: linear plot of sample distribution. D–F: Response to 20 Hz stimulations at juvenile [white dots] and adult [black dots] Thalamo-LA synapses. Same presentation as in A–C.
(0.17 MB TIF)

Figure S3 Decrease in Cortico-LA synaptic strength in adult Mecp2308/Y mice. A: Average Input/output relationships at Cortico-LA synapses of adult WT and KO animals. Ratio between KO/WT data is presented in A2, allowing to better visualize that the decrease of KO response is constant at every stimulation intensity. B: Minimal stimulations at Cortico-LA synapses. B1: example traces showing minimal responses following external capsule stimulations. Typically with the repetitive stimulation of single axons, the rate of successful stimulation increase at a second stimulation applied with a 50 millisecond interval. This is better appreciated in the cumulative plot presented in B2 (same data set). C: Minimal responses at WT and KO Cortico-LA synapses. C1: Typical recordings. C2: cumulative plot showing amplitudes of evoked minimal EPSCs including stimulation failures. C3: The mean amplitude of minimal EPSCs is decreased in Mecp2308/Y adult mice. *: P<0.05. Number of recorded cells is indicated.
(0.29 MB TIF)

Figure S4 Effect of AC activation onto adult Mecp2X/Y and Mecp2308/Y Cortico-LA synapses. A: A 10 minute application of the AC activator Forskolin (FSK, 50 µM) potentiates the Cortico-LA EPSC in young WT animals. B: The same protocol is inefficient in adult WT mice (B1) and Mecp2308/Y mice (B2). C: Summary plot of similar pharmacological experiments in WT and KO mice. ***: P<0.001. Number of recorded cells is indicated.
(0.21 MB TIF)

Figure S5 Extraction of NMDA/AMPA ratio at excitatory projections to the LA. Role of MeCP2 in NMDA/AMPA ratio maintenance during development at Cortico-LA synapses. A1: Postsynaptic currents recorded at various membrane potentials [left], in presence of a GABAA blocker [100 µM Picrotoxin]. Two parameters were analyzed: the peak current between 5–15 msec after the stimulation [grey circles] and the current at 100 msec after the onset of the AMPA response [white circle]. A2: Values from different cells were averaged and displayed typical I/V relationships of AMPA [grey circles] and NMDA currents [white circles]. B1-2: Developmental time course of NMDA/AMPA ratio at Cortico-LA synapses of MeCP2308/Y KO mice [B2] and their WT littermates [B1] Light circles represent single experiments and white dots are mean [± SEM] values at a given post-natal age. C: Bar graphs displaying values of NMDA/AMPA ratio at adult Cortico-LA and Thalamo-LA synapses in MeCP2308/Y KO mice and their WT littermates. *: p<0.05.
(0.17 MB TIF)

Figure S6 Spontaneous Inhibitory transmission in presence and absence of MeCP2. A: Typical recordings of spontaneous IPSCs in adult LA principal cells [recorded at -10mV]. Right: the application of the GABAA receptor blocker Picrotoxin completely blocked outward currents. Scale bars: 20 pA and 1 sec. B: Typical recordings in WT and KO cells. Scale bars: 20 pA and 1 sec. C1: Extracted single IPSCs [20 traces each] are similar in WT and KO cells. Black line: mean current time course. Scale bars: 20 pA and 15 msec. C2: Bar graphs displaying values of sIPSC frequency and amplitude in LA principal cells in adult MeCP2308/Y KO mice and their WT littermates. Number of recorded cells is indicated. In brief, sIPSCs were automatically detected by a template-based routine in the ClampFit 10.0 software. Events were then fitted by a standard bi-exponential equation to extract un-noisy amplitude. In general, sIPSC frequency was determined by visual detection [number of peaks] within a 30 sec recording time period.
(0.25 MB TIF)
