# Supplementary figures and images for: Synaptic Maturation at Cortical Projections to the Lateral Amygdala in a Mouse Model of Rett Syndrome
Source: PLoS One. 2010 Jul 2;5(7):e11399. doi: 10.1371/journal.pone.0011399 (PMC2896423; doi:10.1371/journal.pone.0011399)

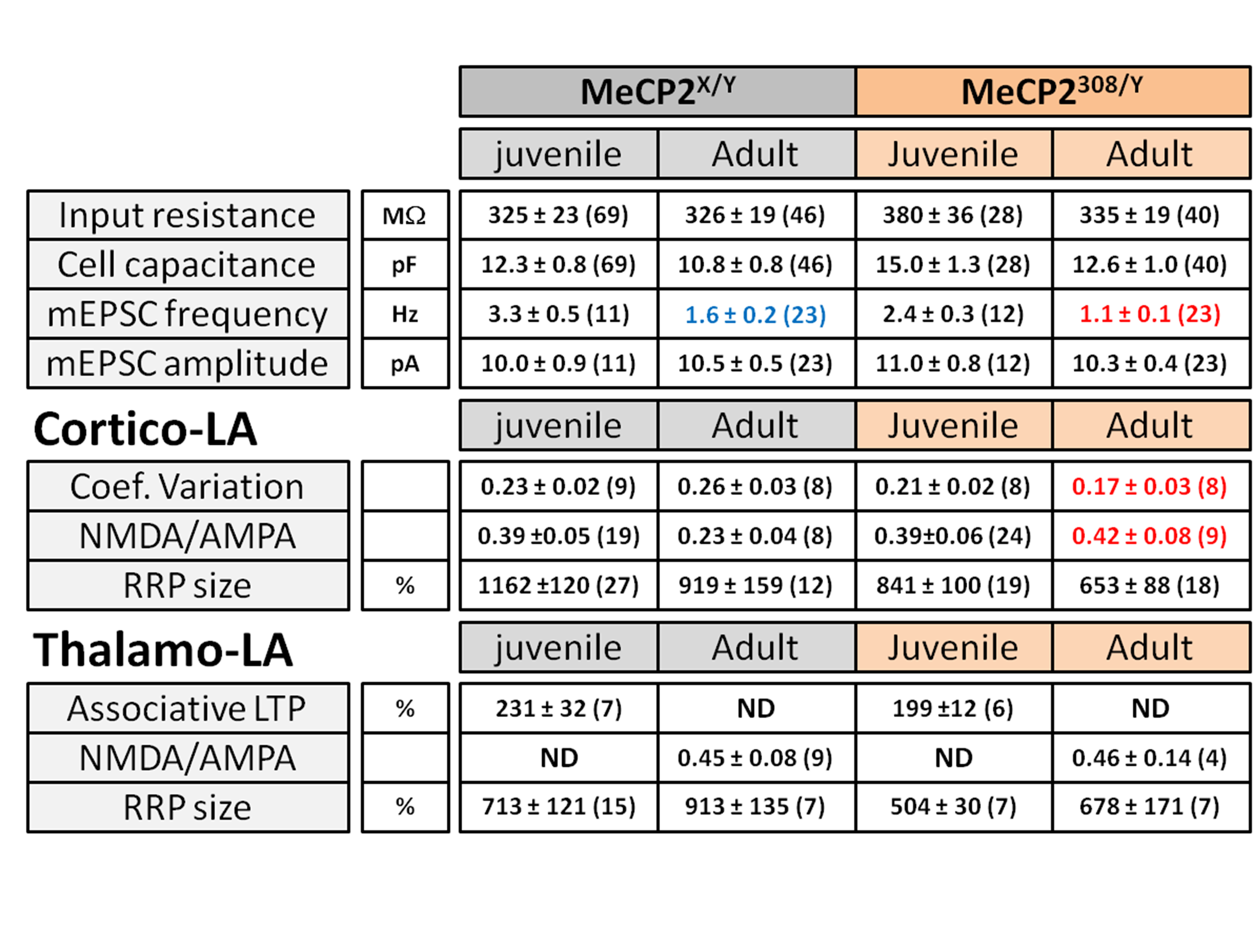

Supplement: Table S1 — Top: Basic cellular and recording parameters were obtained in juvenile and adult WT and KO mice. The unit of a particular measurement is indicated on the left, and the number of recorded cells in brackets. A blue text means a difference between juveniles and adult WT, and red text between WT and KO at a given postnatal age. Methods table S1 The input resistance (Rinput) and cell capacitance (tau/Rinput) were extracted from 10 mV hyper-polarizing steps in voltage clamp mode. Miniature EPSCs (mEPSCs) were automatically detected by a template-based routine in the ClampFit 10.0 software. Events were then fitted by a standard bi-exponential equation to extract un-noisy amplitude. In general, mEPSC frequency was determined by visual detection (number of peaks) during a 0,5 to 10 minute period depending on the event frequency (a minimum of 200 events was analyzed). The coefficient of variation is determined using at least 30 consecutive EPSCs. No difference between mean EPSC amplitude was detected between considered groups. (2.92 MB TIF) [file pone.0011399.s001.tif]

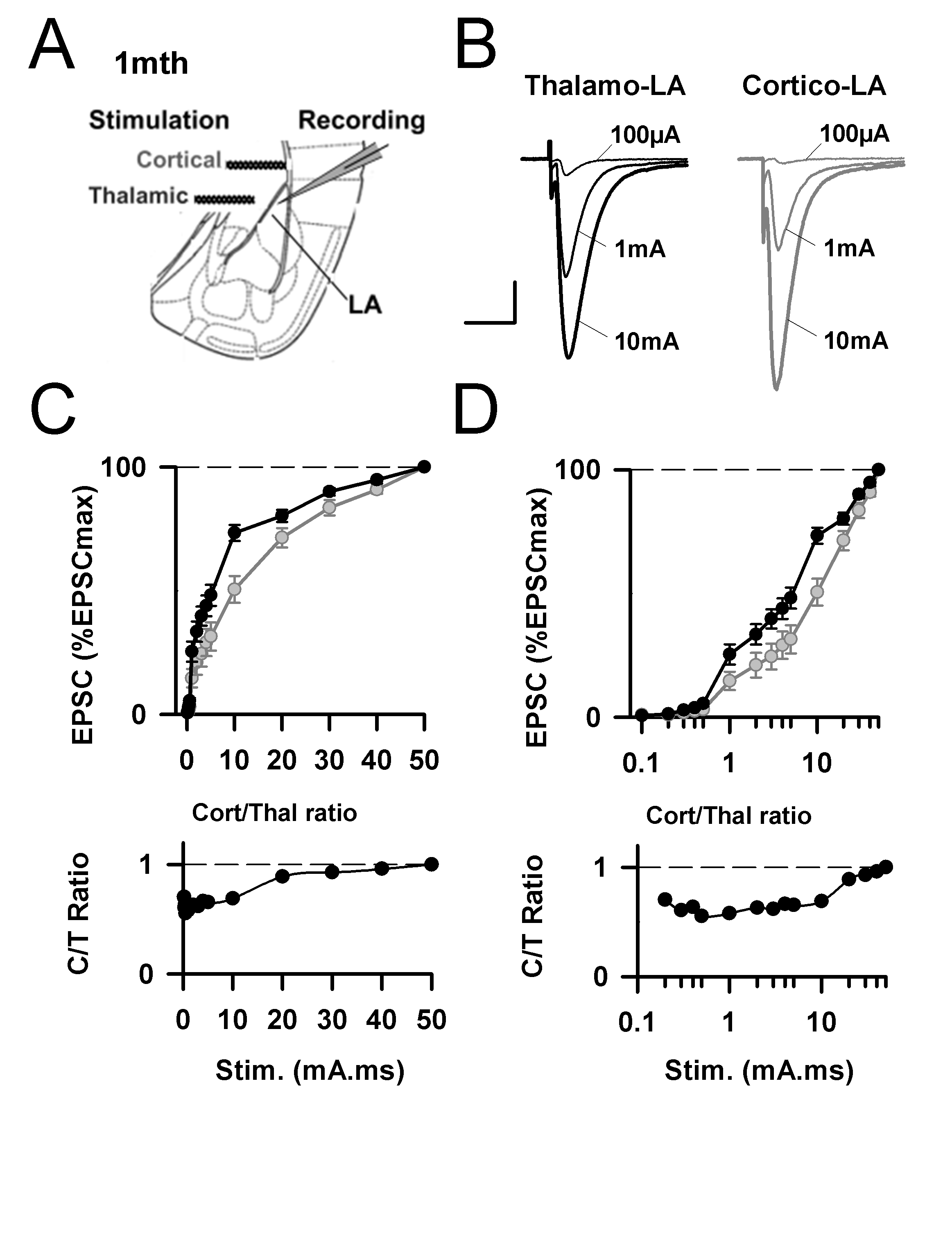

Supplement: Figure S1 — Input/Output curves at Thalamo-LA and Cortico-LA synapses. A: Scheme of the experimental preparation. B: Typical EPSCs recorded in LA principal cells following stimulation in the internal [Thalamo-LA] and external [Cortico-LA] capsules in juvenile animals. Stimulation intensities are indicated. Scale bars: 400 pA and 20 msec. C and D: Top: EPSC amplitude obtained at a given stimulation intensity were average and scaled to the maximal EPSC [at 50mA/msec stimulation]. Grey dots: Cortico-LA EPSCs, Black dots: Thalamo-LA EPSCs. In D, stimulation intensities were presented as a logarithmic function to better visualize the difference in efficacy at low stimulations. Bottom: The ratio between normalized EPSCs is presented, showing that Thalamo-LA synapses are more easily activated than Cortico-LA synapses [ratio <1]. (1.10 MB TIF) [file pone.0011399.s002.tif]

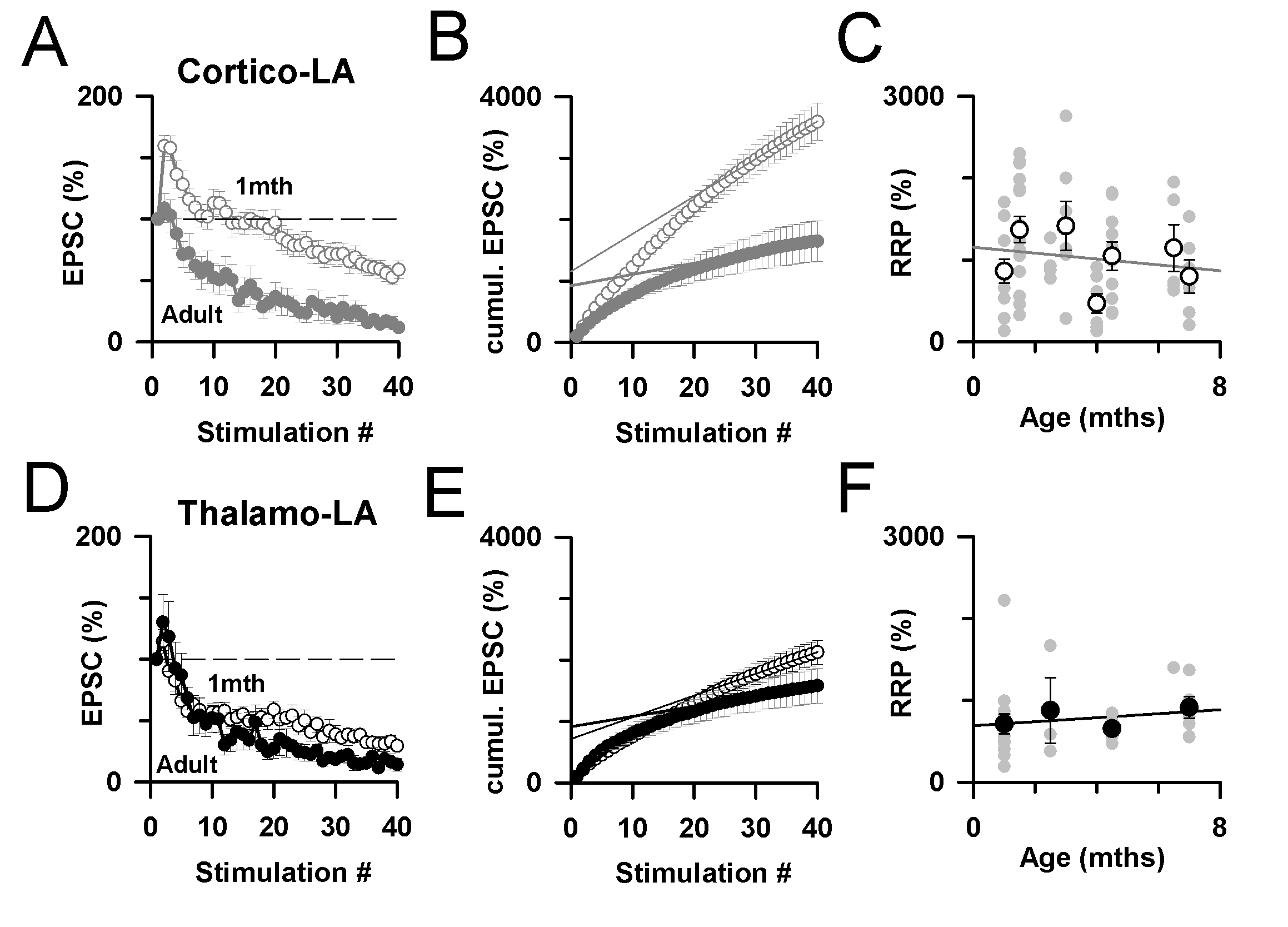

Supplement: Figure S2 — Extraction of RRP size and Refilling rate at excitatory projections to the LA. A-C: Response to 20 Hz stimulations at juvenile [white dots] and adult [grey dots] cortico-LA synapses. A: EPSC amplitude at a given position [#1–40] during 20 Hz trains were averaged [n = 27 and 12 cells respectively]. B: Cumulative EPSC amplitude during 20 Hz trains in juvenile [white dots] and adult [grey dots] cortico-LA synapses. Same data as in A. Linear fit were obtained from #30–40. Its slope is an index of the refilling rate, whereas its extension at y = 0 give the size of the readily releasable pool [RRP]. For further details, see [27]. C: Developmental time course of RRP size at Cortico-LA synapses. Grey circles represent single experiments and white dots are mean [± SEM] values at a given post-natal age. Grey line: linear plot of sample distribution. D–F: Response to 20 Hz stimulations at juvenile [white dots] and adult [black dots] Thalamo-LA synapses. Same presentation as in A–C. (0.17 MB TIF) [file pone.0011399.s003.tif]

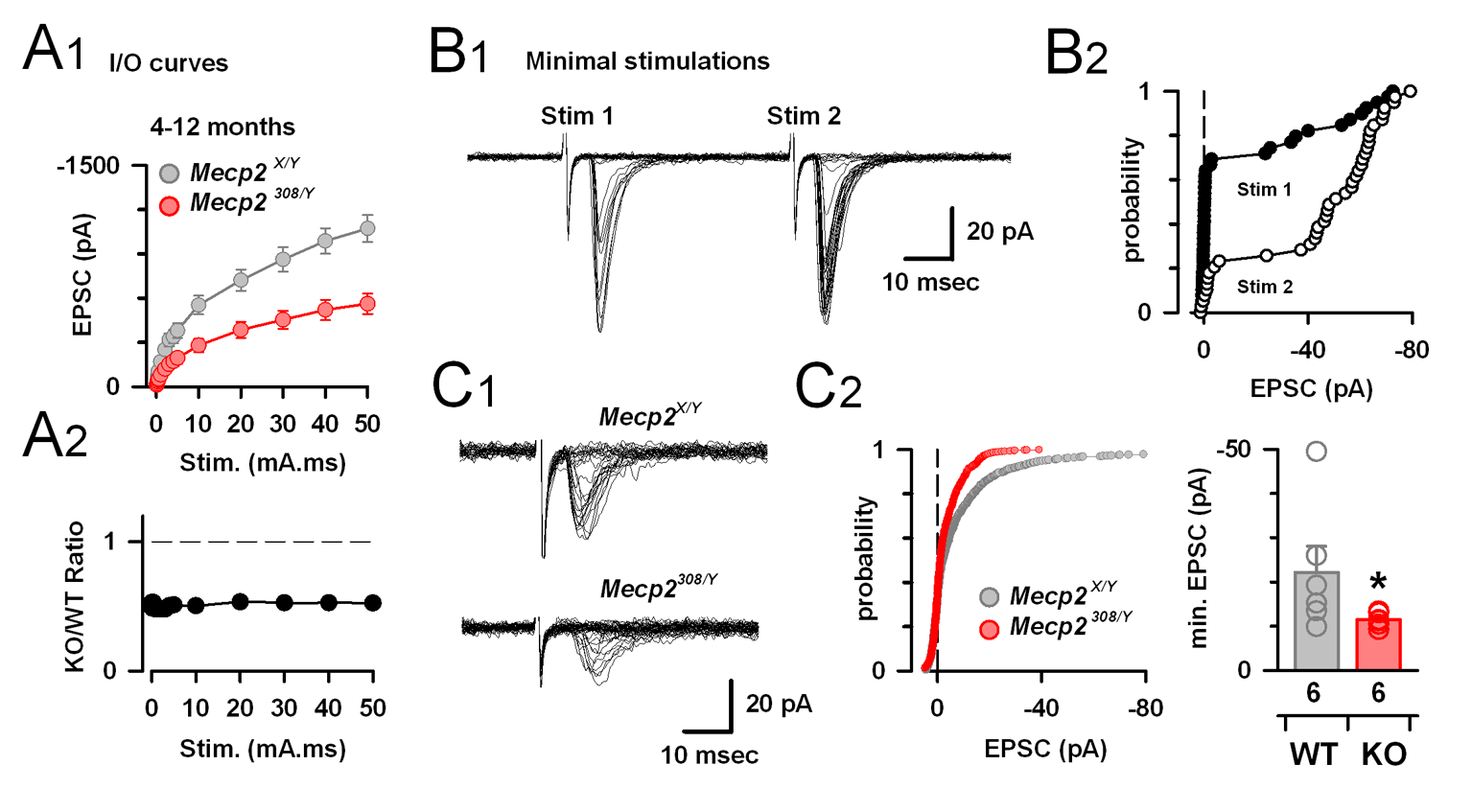

Supplement: Figure S3 — Decrease in Cortico-LA synaptic strength in adult Mecp2308/Y mice. A: Average Input/output relationships at Cortico-LA synapses of adult WT and KO animals. Ratio between KO/WT data is presented in A2, allowing to better visualize that the decrease of KO response is constant at every stimulation intensity. B: Minimal stimulations at Cortico-LA synapses. B1: example traces showing minimal responses following external capsule stimulations. Typically with the repetitive stimulation of single axons, the rate of successful stimulation increase at a second stimulation applied with a 50 millisecond interval. This is better appreciated in the cumulative plot presented in B2 (same data set). C: Minimal responses at WT and KO Cortico-LA synapses. C1: Typical recordings. C2: cumulative plot showing amplitudes of evoked minimal EPSCs including stimulation failures. C3: The mean amplitude of minimal EPSCs is decreased in Mecp2308/Y adult mice. *: P<0.05. Number of recorded cells is indicated. (0.29 MB TIF) [file pone.0011399.s004.tif]

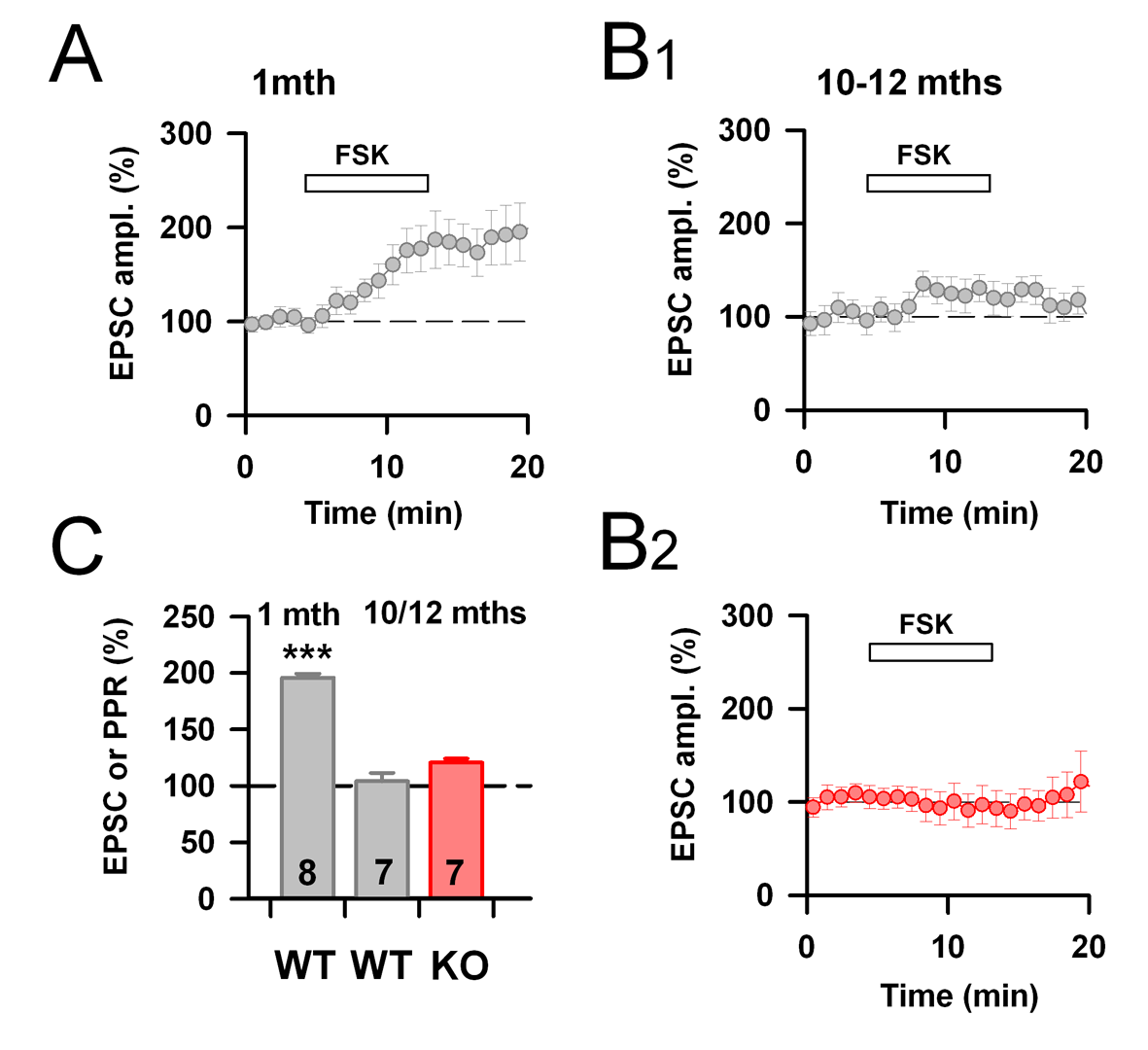

Supplement: Figure S4 — Effect of AC activation onto adult Mecp2X/Y and Mecp2308/Y Cortico-LA synapses. A: A 10 minute application of the AC activator Forskolin (FSK, 50 µM) potentiates the Cortico-LA EPSC in young WT animals. B: The same protocol is inefficient in adult WT mice (B1) and Mecp2308/Y mice (B2). C: Summary plot of similar pharmacological experiments in WT and KO mice. ***: P<0.001. Number of recorded cells is indicated. (0.21 MB TIF) [file pone.0011399.s005.tif]

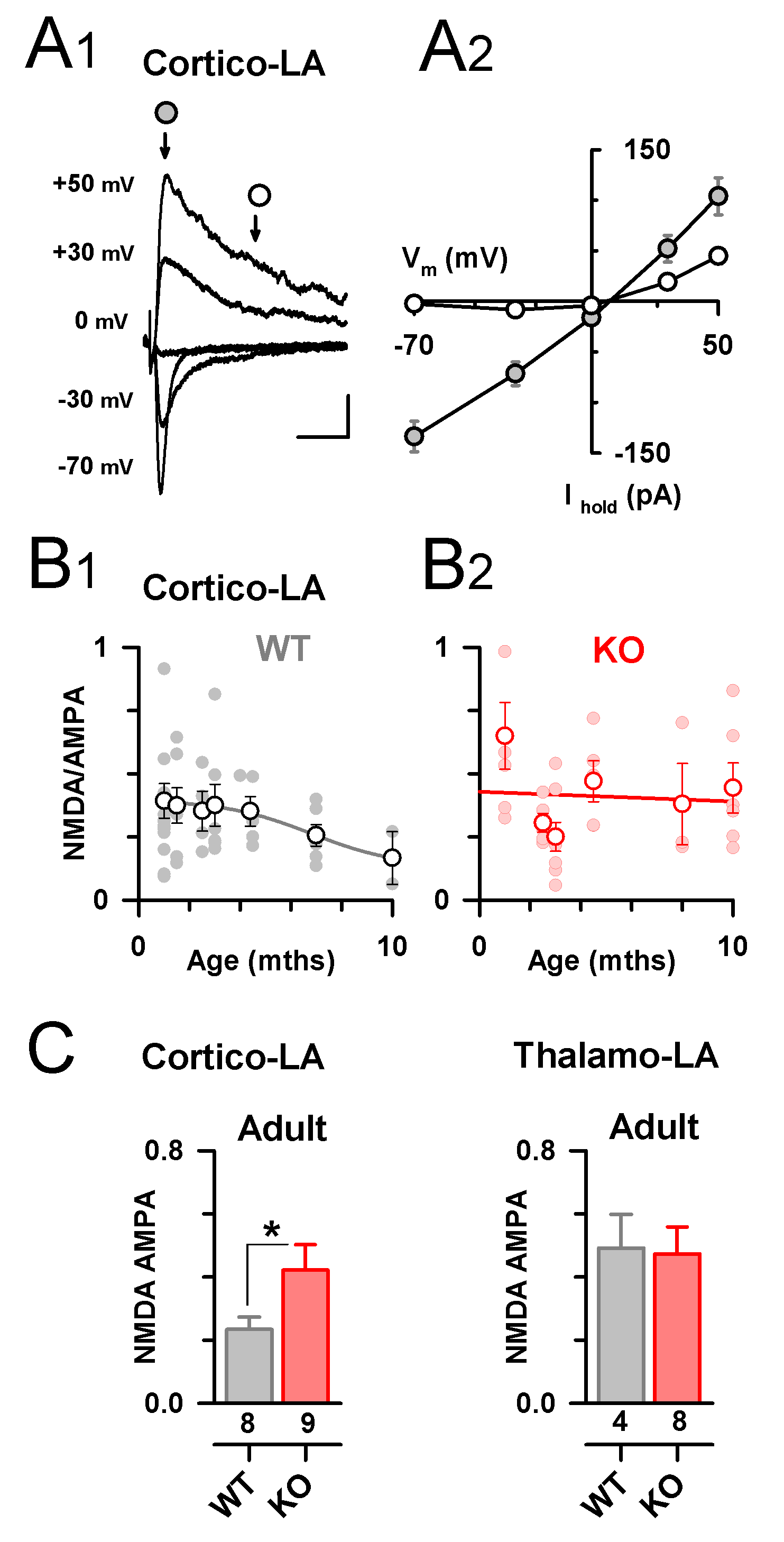

Supplement: Figure S5 — Extraction of NMDA/AMPA ratio at excitatory projections to the LA. Role of MeCP2 in NMDA/AMPA ratio maintenance during development at Cortico-LA synapses. A1: Postsynaptic currents recorded at various membrane potentials [left], in presence of a GABAA blocker [100 µM Picrotoxin]. Two parameters were analyzed: the peak current between 5–15 msec after the stimulation [grey circles] and the current at 100 msec after the onset of the AMPA response [white circle]. A2: Values from different cells were averaged and displayed typical I/V relationships of AMPA [grey circles] and NMDA currents [white circles]. B1-2: Developmental time course of NMDA/AMPA ratio at Cortico-LA synapses of MeCP2308/Y KO mice [B2] and their WT littermates [B1] Light circles represent single experiments and white dots are mean [± SEM] values at a given post-natal age. C: Bar graphs displaying values of NMDA/AMPA ratio at adult Cortico-LA and Thalamo-LA synapses in MeCP2308/Y KO mice and their WT littermates. *: p<0.05. (0.17 MB TIF) [file pone.0011399.s006.tif]

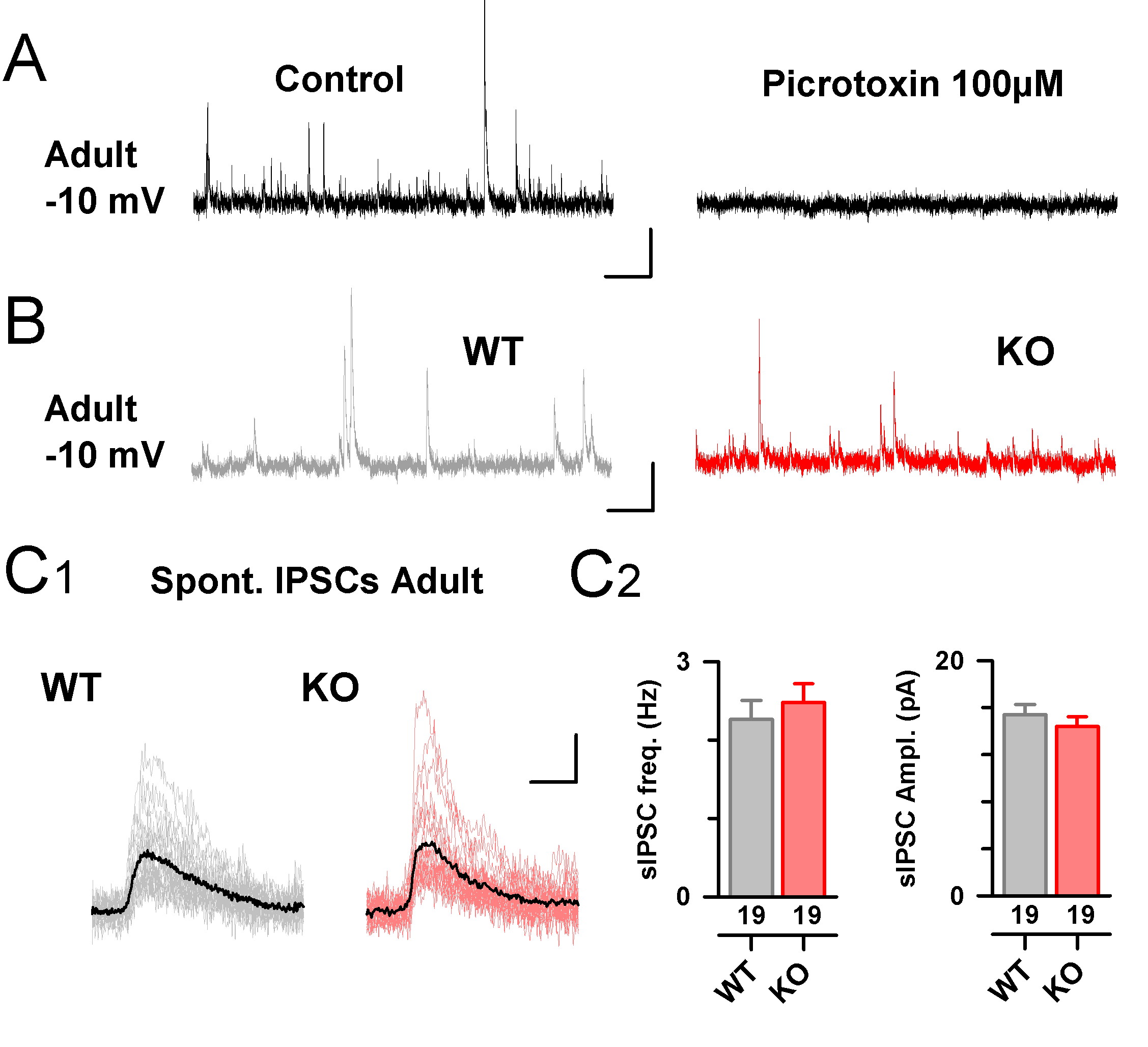

Supplement: Figure S6 — Spontaneous Inhibitory transmission in presence and absence of MeCP2. A: Typical recordings of spontaneous IPSCs in adult LA principal cells [recorded at -10mV]. Right: the application of the GABAA receptor blocker Picrotoxin completely blocked outward currents. Scale bars: 20 pA and 1 sec. B: Typical recordings in WT and KO cells. Scale bars: 20 pA and 1 sec. C1: Extracted single IPSCs [20 traces each] are similar in WT and KO cells. Black line: mean current time course. Scale bars: 20 pA and 15 msec. C2: Bar graphs displaying values of sIPSC frequency and amplitude in LA principal cells in adult MeCP2308/Y KO mice and their WT littermates. Number of recorded cells is indicated. In brief, sIPSCs were automatically detected by a template-based routine in the ClampFit 10.0 software. Events were then fitted by a standard bi-exponential equation to extract un-noisy amplitude. In general, sIPSC frequency was determined by visual detection [number of peaks] within a 30 sec recording time period. (0.25 MB TIF) [file pone.0011399.s007.tif]
